# Pharmaceutical and Medical Uses of Glycerine

**Published:** 1873-02

**Authors:** 


### ARTICLE VIII.

### *Pharmaceutical and Medical Uses of Glycerine.*

Professor Charles A. Joy gives the following summary in the *Journal of Applied Chemistry:*

The following recipe is for the preparation of glycerin lotion: Glycerin, 3 fluid ounces; mucilage quince seeds, U. S. D., 10 fluid drachms; pulverized cochineal, 5 grains; hot water, 1 1-2 fluid ounces; deodorized alcohol, 2 1-2 fluid ounces; oil rose, 8 drops; pulverized gum arabic, a half drachm; water, 8 fluid ounces. Rub the powdered cochineal first, with the hot water gradually added, and then add the alcohol. Triturate the oil of rose with the powdered gum arabic, and gradually add the water, as in making an emulsion. With this mix well the solution first formed, and filter, and to the filtered liquid add the glycerin and mucilage of quince seeds, and shake well. The mucilage of quince seeds should always be freshly made.

A good ointment is made by boiling 80 grains of starch in one fluid ounce of glycerin. The ointment never becomes rancid; it is inodorous, and does not change. Corn starch has been found best suited for the purpose. A stiff plaster can also be made with 150 grains of starch boiled in one ounce of glycerin. A sedative plaster is made with sulphate of atropia, 3 grains; veratria, 3 grains; sulphate morphia, 8 grains; otto of roses, 1 drop; hard glycerin ointment, 1 ounce.

Four parts by weight of yolk of egg rubbed in a mortar with parts of glycerine, gives you a preparation of great value as an unguent for application to broken surfaces of the skin. It has a honey-like consistency, is unctuous, like fatty substances, but has the advantage over them of being easily removed by water. It is also unalterable. Applied to the skin it forms a varnish, which effectually excludes the air and prevents its irritating effects. These properties render it serviceable for erysipelas and cutaneous diseases, of which it allays the action.

Glycerine vaccine lymph is highly prized on account of its stable character. For its preparation, the pustules of a healthy vaccinated person are opened with a needle and the effluent carefully removed by means of a lancet. It is put into a suitable vessel and mixed with twice its quantity of chemically prepared glycerin and as much distilled water. The liquids must be thoroughly incorporated by means of a paint brush, and for preparation can be put into capillary tubes.

A glycerine ointment of much repute for chapped hands and excoriations is made as follows: One half ounce ot spermaceti is melted together with a drachm of white wax and two fluid ounces of oil of almonds, by a moderate heat; the mixture is poured into a Wedgewood mortar, when a fluid ounce of glycerin is added to it and rubbed until the ingredients are thoroughly mixed and cold.

The introduction of glycerin in small quantity into pills prevents induration and decomposition. Where resin is present it is well to add a small quantity of alcohol. In general the substitution of glycerin for syrups, in prescribing medicine in liquid form is highly recommended by physicians; the reasons for this recommendation may be stated as follows: It possesses greater solvent power, and mixes well with most substances; it acts as a preservative to the medicine by preventing fermentation and decomposition; in the practice of children it counteracts fermentation in the stomach, acts as a nutritive, and diminisher irritation in the alimentary canal; it has no superior for giving acrid substances, such as tincture of guaiac, turpentine, ammonia, chloroform, acids, etc.

Sesquichloride of iron and glycerin have been prescribed in cases of diptheria. The mixture consists of two ounces of pure glycerin and 20 drops of the liqnor ferri sesquichloride. Half a teaspoonful is given every hour throughout the day and night until the symptoms appear to be mitigated. With the object of dissolving the exudate, a mixture of two ounces of glycerine and 20 grains of borax is similarly given in doses of a teaspoonful at a time. The good effects in cases of hourseness and loss of voice led to the application of glycerin to the treatment of croup. The application is made by inhalation through some form of atomizing apparatus.

Dr. Fanto (*Wiener Medicinische Zeitung*) remarks that leading dermatologists are coming to use glycerin, locally applied, as a substitute for many internal remedies that have been extensively employed in cutaneous affections. Glycerin has proved especially valuable in cases of abnormal secretion of sebaceous substances. This is caused by disease of the sebaceous glands, and has its seat not, as was formerly supposed, in the subcutaneous connective tissue, but in the corium itself. Sometimes the complaint takes the form of hypersecretion from these glands. This occurs for the most part in infancy, and is known as seborrhœa. It is most commonly seen on the scalp, on the face near the ear muscles, and more rarely on the extremities. In such cases glycerine acts excellently in softening the hardened masses of sebum on the surface of the skin, and in diminishing the irritation of the organs affected. In conjunction with borax, zinc and acetate of lead, it also diminishes the amount of secretion. In many instances the treatment must be continued for a considerable time in order to effect a cure.

Glycerin is equally useful in cases where there is a diminution of the sebaceous secretion, which may lead to pityriasis. In this harsh state of the skin, the softness and natural elasticity may be restored by rubbing glycerin into it. None but a perfectly pure article should be used.—*Canadian Pharmaceutical Journal.*

---

